# Socioeconomic Differences in SARS-CoV-2 Infection and Vaccination in Germany: A Seroepidemiological Study After One Year of COVID-19 Vaccination Campaign

**DOI:** 10.3389/ijph.2023.1606152

**Published:** 2023-09-14

**Authors:** Susanne Bartig, Florian Beese, Benjamin Wachtler, Markus M. Grabka, Elisabetta Mercuri, Lorenz Schmid, Nora Katharina Schmid-Küpke, Madlen Schranz, Laura Goßner, Wenke Niehues, Sabine Zinn, Christina Poethko-Müller, Lars Schaade, Claudia Hövener, Antje Gößwald, Jens Hoebel

**Affiliations:** ^1^ Department of Epidemiology and Health Monitoring, Robert Koch Institute, Berlin, Germany; ^2^ Socio-Economic Panel, German Institute for Economic Research, Berlin, Germany; ^3^ Department of Infectious Disease Epidemiology, Robert Koch Institute, Berlin, Germany; ^4^ Institute for Employment Research (IAB), Nuremberg, Germany; ^5^ Research Centre of the Federal Office for Migration and Refugees (BAMF-FZ), Nuremberg, Germany; ^6^ Centre for Biological Threats and Special Pathogens, Robert Koch Institute, Berlin, Germany

**Keywords:** SARS-CoV-2, COVID-19, vaccination, socioeconomic position, seroepidemiology, RKI-SOEP-2

## Abstract

**Objective:** To evaluate the socioeconomic patterns of SARS-CoV-2 antigen contacts through infection, vaccination or both (“hybrid immunity”) after 1 year of vaccination campaign.

**Methods:** Data were derived from the German seroepidemiological Corona Monitoring Nationwide study (RKI-SOEP-2; *n* = 10,448; November 2021–February 2022). Combining serological and self-report data, we estimated adjusted prevalence ratios (PR) of SARS-CoV-2 infection, COVID-19 vaccination, basic immunization (at least two SARS-CoV-2 antigen contacts through vaccination and/or infection), and three antigen contacts by education and income.

**Results:** Low-education groups had 1.35-times (95% CI 1.01–1.82) the risk of SARS-CoV-2 infection compared to high-education groups. COVID-19 vaccination (at least one dose) and basic immunization decreased with lower education and income. Low-education and low-income groups were less likely to have had at least three antigen contacts (PR low vs. high education: 0.74, 95% CI 0.65–0.84; PR low vs. high income: 0.66, 95% CI 0.57–0.77).

**Conclusion:** The results suggest a lower level of protection against severe COVID-19 for individuals from low and medium socioeconomic groups. Pandemic response and vaccination campaigns should address the specific needs and barriers of these groups.

## Introduction

In early 2020, the newly emerged coronavirus (SARS-CoV-2) began to spread around the world, and on 11th March 2020 the World Health Organization characterized the COVID-19 outbreak as a pandemic [1]. More than 3 years into the pandemic, there is plenty—and still increasing—international evidence to suggest that socioeconomically disadvantaged groups have a higher risk of infection with SARS-CoV-2 [2]. However, these socioeconomic inequalities in infections have changed during different phases of the pandemic [3]. In Germany, the virus emerged in spring 2020 predominantly among residents of more affluent regions. Soon afterwards, higher infection rates shifted to socioeconomically more deprived regions [4]. This pattern of changing socioeconomic inequalities in SARS-CoV-2 infection was also observable during the subsequent infection waves in Germany [5]. Considered cumulatively over the first two pandemic waves, people with a low socioeconomic position (SEP) had an up to twofold higher risk for SARS-CoV-2 infection than those with a high SEP [6]. Evidence from various countries also showed higher risks of severe disease progression such as COVID-19-related hospitalization [7, 8] and mortality [9, 10] in groups with a lower SEP. These inequalities might, to a certain degree, be attributable to unequal infection risks or greater vulnerability due to higher prevalences of comorbidities [11]. Contrarily, one might argue that higher infection rates during the early phases of the pandemic also mean that those who survived the infection were possibly better protected against severe COVID-19 after a subsequent SARS-CoV-2 infection due to the immunological response to their first antigen contact [12]. This may have been particularly relevant during the pre-vaccine phases of the pandemic, but changed after effective vaccines against COVID-19 became available.

COVID-19 vaccination aims to reduce severe courses of the disease and deaths and to prevent the transmission of SARS-CoV-2 [13]. In Germany, the COVID-19 vaccination campaign began in late December 2020 with BioNTechPfizer’s Comirnaty^®^ vaccine. Up until February 2023, six additional vaccines were approved in Germany [14]. However, because the vaccines’ availability was initially limited, some groups were prioritized at the beginning of the German vaccination campaign: In addition to individuals with an elevated risk of severe COVID-19, e.g., due to their age or other risk factors, priority was primarily given to people with a high occupational risk of exposure to SARS-CoV-2, such as healthcare workers. Exclusive access for prioritized groups was abolished in June 2021, 6 months after the start of the vaccination campaign. Since then, everyone aged 18 years and older has had the opportunity to be vaccinated against COVID-19. In December 2021, a booster dose was officially recommended in Germany by the National Immunization Technical Advisory Group (NITAG) for people above the age of 18 [13]. The vaccination against COVID-19 in Germany, and in most countries worldwide, was not mandatory but a free individual choice and free of charge. However, there were several administrative and legal restrictions for unvaccinated individuals, e.g., when travelling. After the data-collection period, mandatory vaccination for employees working in specific institutions such as nursing homes was temporarily introduced in Germany. Various studies, internationally and for Germany, indicate socioeconomic differences in willingness to be vaccinated and in COVID-19 vaccination uptake during the first months of vaccinations [15–17].

Research suggests that at least three SARS-CoV-2 antigen contacts through vaccination and/or infection may effectively protect against severe COVID-19, in particular through “hybrid immunity” [12, 18]. However, evidence on the association between SEP and the risk of infection with SARS-CoV-2, especially in later pandemic phases, COVID-19 vaccination and ‘hybrid immunity’ from both infection and vaccination is still scarce for Germany. This study aims to evaluate the socioeconomic patterning of different constellations of SARS-CoV-2 antigen contacts from infection and vaccination within the German adult population. It thus investigates how 1) SARS-CoV-2 infections, 2) COVID-19 vaccinations, and hence 3) possible protection against SARS-CoV-2 infection and a severe course of COVID-19 are socioeconomically distributed—2 years into the pandemic and after 1 year of the vaccination campaign in Germany.

## Methods

### Data and Study Design

The data come from the seroepidemiological “Corona Monitoring Nationwide (RKI-SOEP-2)” study, a cooperative project involving the Robert Koch Institute (RKI), the Socio-Economic Panel (SOEP) at the German Institute for Economic Research (DIW Berlin), the Institute for Employment Research (IAB), and the Research Centre of the Federal Office for Migration and Refugees (BAMF-FZ). The study collected 1) dried blood spot (DBS) samples for the detection of IgG antibodies against SARS-CoV-2 and 2) data from a self-administered questionnaire. The study was hosted in the SOEP, which is a German nationwide dynamic cohort based on population-based random samples, which allows representative statements about people in private households in Germany [19]. The gross sample of the RKI-SOEP-2 study comprised all persons aged 14 and older who participated in the SOEP survey wave in 2021. Data collection took place from November 2021 to March 2022. All SOEP households in the gross sample were invited to participate in the study. An invitation packet was sent to each target person, containing both an individual invitation and the study materials (e.g., questionnaire, blood self-sampling kit for capillary blood). Respondents could fill in the self-administered questionnaire either in paper form or online. The questionnaire covered topics such as experienced SARS-CoV-2 infections, COVID-19 vaccination status and willingness to be vaccinated, health status, and health behaviours. To increase participation in the study, a post-paid monetary incentive (10 euros for adults, 5 euros for adolescents) was announced. In addition, the participants who returned dried blood samples (96.2%) received written notification of their laboratory results. All participants gave their written informed consent to participate in the study. The study was approved by the Ethics Committee of the Berlin Chamber of Physicians (Eth-33/20) in compliance with the Declaration of Helsinki. A detailed study description can be found elsewhere [20].

### Infection, Vaccination and Serostatus

We defined SARS-CoV-2 infection and COVID-19 vaccination status based on individual self-reports and serological assays for SARS-CoV-2 antibodies. Self-reported SARS-CoV-2 infection was assessed via the survey question “Have you ever been infected with the coronavirus (SARS-CoV-2) detected by a PCR-test (yes, no, do not know)?”. If the question was answered with yes, the self-reported data were used to define participants as previously infected. If the question was answered with no, seropositivity data were considered. With a positive test for anti-N antibodies (which are not produced after vaccination) the participant was defined to be previously infected. Finally, if the participant reported no vaccination against SARS-CoV-2, seropositivity for anti-S-antibodies was defined as previous infection. If none of the three definitions was met, the participant was defined as not infected. The Euroimmun ELISAs (enzyme-linked immunosorbent assays) anti-SARS-CoV-2-QuantiVac and anti-SARS-CoV-2-NCP were used, respectively, for the detection of anti-S (S1 domain of the spike protein) and anti-N (nucleocapsid protein, NCP) antibodies in dried blood samples. To determine seropositivity for anti-N antibodies in dried blood samples, the ratio cut point for serum samples provided by the manufacturer was adapted from 1.1 to 0.95 (sensitivity: 90.2%, specificity: 95.3%) according to a validation study. Details on this study can be found elsewhere [21]. In this analysis, “having being vaccinated” means having received at least one dose of any COVID-19 vaccine (self-reported). Basic immunization was defined as having had any of at least two self-reported vaccine doses, or a combination of at least one self-reported vaccine dose and a previous infection (self-reported or seropositivity for anti-N). Regardless of their chronological order, we referred to a combination of infection and vaccination as “hybrid immunity.” Three exposures to SARS-CoV-2 were defined as either having three self-reported vaccine doses, or a combination of at least two self-reported vaccine doses and a previous infection. A detailed operationalization of the outcomes can be found elsewhere [22].

### Socioeconomic Position

SEP was measured via two socioeconomic indicators using education data from the 2020 SOEP wave (or the latest available data from earlier waves) and income data from 2021. According to the 2011 version of the International Standard Classification of Education (ISCED), participants’ highest school and vocational qualifications were classified as low (lower secondary education or below), medium (upper secondary or post-secondary education) and high (tertiary education) [23]. Equivalized monthly disposable household income was calculated by dividing the household’s total disposable income by the square root of the number of household members [24] and categorized into low (quintile 1), middle (quintiles 2–4) and high (quintile 5).

### Statistical Analysis

Descriptive results are presented as prevalence with 95% confidence intervals (95% CI) for each outcome of the two dimensions of SEP considered in this analysis. Socioeconomic differences in the temporal development of vaccination coverage during the vaccination campaign were estimated by Kaplan-Meier analysis using self-reports on the date of first vaccination. The association between SEP indicators and SARS-CoV-2 infection, COVID-19 vaccination, basic immunization and at least three antigen contacts was estimated by calculating prevalence ratios (PRs) with 95% CI and *p*-values using Poisson regressions with household-clustered standard errors. Adjustments were made for age, sex, migration status, urban–rural residence, federal state and date of participation. PRs by income were additionally adjusted for education since educational attainment is commonly causally anterior to income and thus a potential confounder of the income-outcome associations. In order to consider further potential covariates, we additionally included the household composition (number of household members with differentiation of the presence of children) and the employment status in sensitivity analyses. As people aged 60 years and older were prioritized at the beginning of the vaccination campaign, age-differentiated analyses of the COVID-19 vaccination were also carried out. Furthermore, in order to decompose the outcome at least three antigen contacts, we ran an additional analysis by calculating prevalence estimates and adjusted prevalence ratios for at least three antigen contacts (only vaccine-induced) and at least three antigen contacts (hybrid induced through vaccination and infection) by education and income.

The analyses were calculated with weighting factors to compensate for systematic non-response. The weights resulted from complex non-response modelling at both the household and the individual level and, in addition, adjust the sample to match the official German population statistics by age, sex, citizenship (German vs. non-German), federal state, household type and size, as well as owner-occupied housing [20, 25]. More details of the weighting procedure applied in the RKI-SOEP2- study can be found in Danne et al. [25]. Analyses were restricted to adults aged 18 years and older since the SEP of younger persons cannot be determined in the same way as that of adults.

All analyses were conducted using Stata 17.0 and R version 4.1.2.

## Results

Of the 10,448 participants, the majority (70.2%) participated in November and December 2021 (Table 1). The overall prevalence of SARS-CoV-2 infection was 9.8%. Most of the participants had been vaccinated at least once (94.0%) and had basic immunization (91.0%); one-third (33.2%) had had at least three SARS-CoV-2 antigen contacts.

**TABLE 1:** Characteristics of the study population (*n* = 10,448; Germany, 2021–2022).

	*n*	%^a^
Sex		
Female	5,644	51.1
Male	4,804	48.9
Missing	–	–
Age group (years)		
18–39	2,584	32.1
40–59	4,300	34.1
60+	3,564	33.8
Missing	–	–
Month of participation		
November	4,955	(47.4)
December	2,383	(22.8)
January	2,239	(21.4)
February^b^	871	(8.3)
Missing	–	–
Migration background		
No migration background	8,750	78.6
Direct migration background	1,120	16.0
Indirect migration background	511	5.4
Missing	67	–
Urban/rural residence		
Urban	6,908	70.0
Rural	3,438	30.0
Missing	102	–
Education		
Low	967	10.5
Medium	4,948	53.7
High	4,061	35.8
Missing	472	–
Income		
Low	1,525	20.6
Medium	5,713	60.0
High	2,494	19.4
Missing	716	–
SARS-CoV-2 infection		
Yes	1,100	9.8
No	9,342	90.2
Missing	6	–
COVID-19 vaccination (at least one dose)		
Yes	9,670	94.0
No	608	6.0
Missing	170	–
Basic immunization (infection and/or vaccination)		
Yes	9,439	91.0
No	811	9.0
Missing	198	–
At least 3 SARS-CoV-2 antigen contacts		
Yes	3,958	33.2
No	6,292	66.8
Missing	199	–

*n* = unweighted number of participants.

^a^ Weighted %, unweighted % in brackets, % were calculated without missing values.

^b^ 14 (0.1%) observations included from the beginning of March.

### SARS-CoV-2 Infection

**FIGURE 1:**
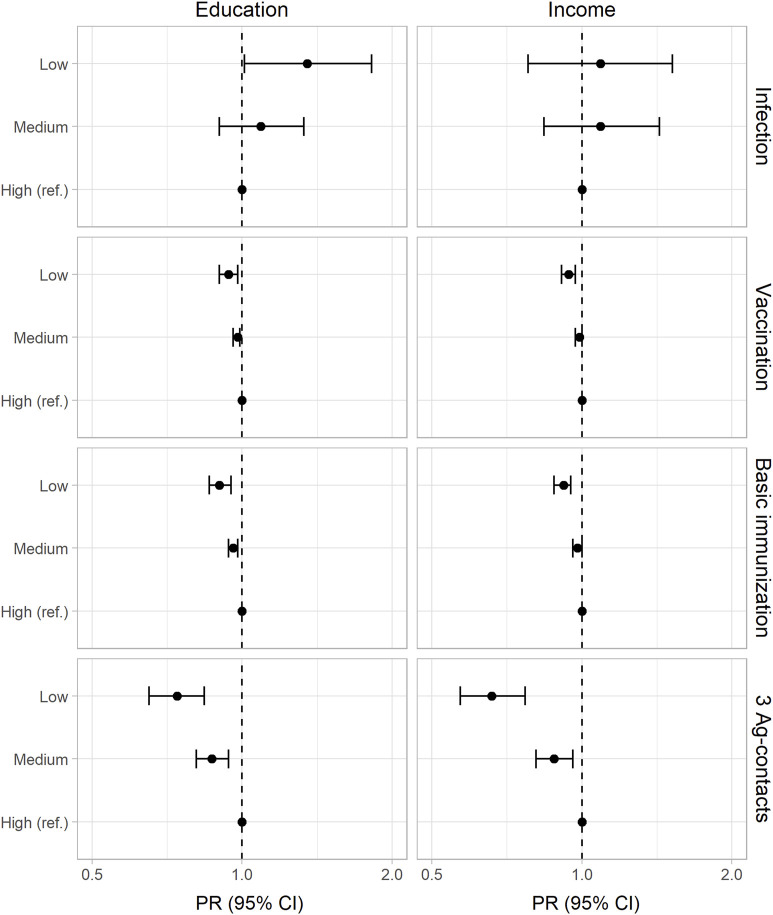
Adjusted prevalence ratios (PR) and 95% confidence intervals (95% CI) for SARS-CoV-2 infection, COVID-19 vaccination (at least one dose), basic immunization, and at least 3 antigen contacts (Germany, 2021–2022). Results from Poisson regression adjusted for age, sex, migration status, urban–rural residence, federal state, date of participation (and education). Exact estimates and *p*-values are given in Supplementary Table S1.

Table 2 shows the prevalence of SARS-CoV-2 infection by each socioeconomic indicator, with higher prevalence rates in lower socioeconomic groups but overlapping 95% CI between all SEP groups. When adjusted for covariates (Figure 1, Supplementary Table S1), income was not associated with infection status (PR: 1.09, 95% CI: 0.78–1.52, *p* = 0.604), whereas people with lower education showed a 35% (PR: 1.35, 95% CI: 1.01–1.82, *p* = 0.045) higher risk of infection than those with higher education. In our sensitivity analysis with additional adjustments for employment status und household composition, the PR estimate altered marginally while remaining significant for education (PR low vs. high: 1.38, 95% CI: 1.01–1.87, *p* = 0.041) and not significant for income (PR low vs. high: 1.30, 95% CI: 0.94–1.82, *p* = 0.115).

**TABLE 2:** Prevalence of SARS-CoV-2 infection, COVID-19 vaccination, basic immunization, and at least three antigen contacts by indicators of socioeconomic position among adults in Germany, 2021–2022.

	SARS-CoV-2 infection	COVID-19 vaccination^a^	Basic immunization^b^	At least 3 antigen contacts
	% (95%-CI)	% (95%-CI)	% (95%-CI)	% (95%-CI)
Education				
Low	12.9 (9.9–16.7)	89.4 (85.3–92.5)	84.4 (79.8–88.1)	28.0 (23.9–32.5)
Medium	9.5 (8.3–10.8)	93.6 (92.5–94.6)	90.6 (89.2–91.8)	32.5 (30.6–34.4)
High	9.3 (7.9–10.9)	95.4 (94.3–96.3)	93.5 (92.1–94.7)	35.3 (33.0–37.6)
Income				
Low	10.7 (8.6–13.3)	89.3 (86.4–91.6)	85.1 (81.8–87.9)	24.0 (20.8–27.4)
Medium	9.9 (8.7–11.3)	94.4 (93.3–95.3)	91.6 (90.3–92.7)	33.1 (31.2–35.1)
High	8.0 (6.3–10.1)	97.3 (95.8–98.3)	95.3 (93.4–96.6)	39.0 (35.8–42.4)

% weighted prevalence; CI, confidence interval.

^a^ At least one dose of COVID-19 vaccine.

^b^ Due to SARS-CoV-2 infection and/or COVID-19 vaccination.

### COVID-19 Vaccination

**FIGURE 2:**
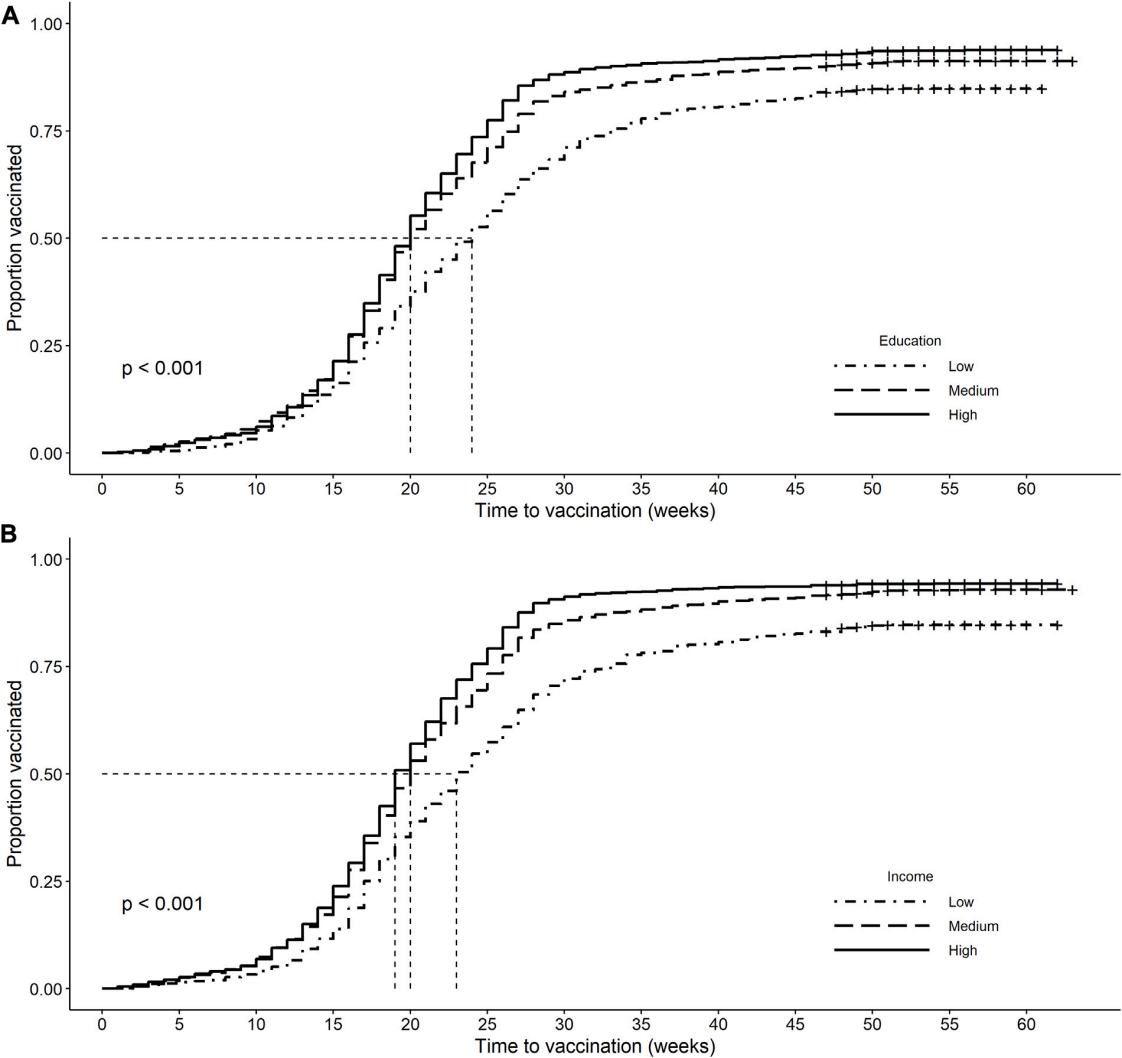
Kaplan-Meier curves of first vaccination from the date of the European Union’s authorization of Comirnaty® (2020-12-21) to the end of the study data collection period (Germany, 2021–2022). Vertical dashed lines indicate median time to first vaccination. Individuals are censored on their survey participation date when no vaccination occurred. Panel **(A)**: Kaplan-Meier curves by education. Median time to vaccination in weeks (95% CI): low: 24 (23, 25), medium: 20 (20, 20), high: 20 (19, 20), log rank: *p* < 0.001. Panel **(B)**: Kaplan-Meier curves by income. Median time to vaccination in weeks (95% CI): low (Q1): 23 (22, 24), medium (Q2–4): 20 (20, 20), high (Q5): 19 (19, 20), log rank: *p* < 0.001.

The prevalence for COVID-19 vaccination differed according to both socioeconomic indicators (Table 2). Low education and income groups were less likely to have received a first dose of vaccine against COVID-19 than those in the high SEP groups. Figure 2 shows the Kaplan-Meier curves for the dates of first vaccination during the period beginning with the European Union’s authorization of Comirnaty^®^ until the end of data collection by education (panel A) and income (panel B). The time to first vaccination differed by both socioeconomic indicators (log rank: *p* < 0.001) with the highest median time to vaccination in the lowest socioeconomic groups.

Results of the adjusted Poisson regressions showed that the lowest education (PR low vs. high: 0.94, 95% CI: 0.90–0.98, *p* = 0.002) and income groups (PR low vs. high: 0.94, 95% CI: 0.91–0.97, *p* < 0.001) had a lower COVID-19 vaccination coverage (Figure 1). Stratifying this analysis by age groups (Supplementary Table S2), educational differences in vaccination coverage resulted in adults under 60 – but not in those aged 60 years and older—being prioritized in the first months of the vaccination campaign. Regarding income, age-differentiated Poisson regressions showed socioeconomic differences (low vs. high income) for both age groups.

### SARS-CoV-2 Antigen Contacts

The proportions of adults with basic immunization and those with at least three antigen contacts increased with higher education and income (Table 2). After adjusting for covariates, education (PR low vs. high: 0.90, 95% CI: 0.86–0.95, *p* < 0.001) and income (PR low vs. high: 0.92, 95% CI: 0.88–0.95, *p* < 0.001) remained positively associated with having basic immunization. Furthermore, the results showed socioeconomic gradients in the prevalence ratios of at least three antigen contacts (Figure 1) among education (PR low vs. high: 0.74, 95% CI: 0.65–0.84, *p* < 0.001) and income groups (PR low vs. high: 0.66, 95% CI: 0.57–0.77, *p* < 0.001). When decomposing having at least three antigen contacts to those that are only vaccine-induced, we found decreasing prevalence estimates with lower education and income. Adjusting for covariates reveals socioeconomic gradients by education and income. For those that are induced through vaccination and infection (hybrid immunity), we found no differences in prevalence estimates and adjusted prevalence ratios (Supplementary Table S3).

## Discussion

After almost 2 years of COVID-19 pandemic and 1 year of the vaccination campaign, adults with lower education were more likely to have been infected with SARS-CoV-2. Also, low and medium SEP groups were less likely to have basic immunization or to have had at least three antigen contacts with SARS-CoV-2. Furthermore, adults with lower SEP were less likely to have been vaccinated and have received their first dose of COVID-19 vaccine later in the campaign. The results indicate that there is still prevention potential in low and medium socioeconomic groups, which together account for the majority of Germany’s population, in order to approach and maintain high protection against severe COVID-19 in the population as a whole.

The educational differences in infection risk with SARS-CoV-2 are in line with previous research from Germany for the first year of the pandemic [6]. A conceptual framework by Bambra [11] proposes unequal exposure (e.g., through opportunities to work from home), unequal transmission (e.g., through housing conditions) and unequal susceptibility (e.g., through comorbidities) as underlying pathways of health inequalities with emerging infectious diseases such as COVID-19. Empirically, individuals with lower SEP were less able to work from home [26], to reduce their contacts during the pandemic [27], and are generally more likely to live in crowded conditions [26]. Moreover, there are educational differences in factors such as risk perception or preventive behaviour regarding COVID-19 [28], which might contribute to a higher infection risk for low education groups. Our sensitivity analysis suggested that the household composition and employment status altered the education-infection association only marginally. This aspect could be investigated in more detail in further research in order to examine the explanatory extent of these and other potential confounding factors such as occupation [29], which was beyond the scope of our analyses.

Regarding COVID-19 vaccination, our results are consistent with previous research indicating socioeconomic differences in COVID-19 vaccination uptake [16]. In addition, several studies show that COVID-19 vaccine hesitancy is also associated with income and education [30, 31]. But there is a lack of studies that examine the reasons for the lower uptake and coverage of COVID-19 vaccination in socioeconomically disadvantaged groups. The internationally established “5C” model suggests several psychological reasons for the different uptake of vaccination in general [32, 33]. Research on this model shows that confidence (e.g., in the safety and effectiveness of the vaccine), constraints (structural and psychological barriers), complacency (perceived risk of the disease), calculation (extent of information searching, risk-benefit analysis) and collective responsibility (willingness to protect others) influence COVID-19 vaccination propensities [34–36]. Further research is needed to explore to what extent these five psychological antecedents of vaccination uptake contribute to explaining socioeconomic differences in COVID-19 vaccination prevalence. Misinformation about COVID-19, often from social media, reduces confidence in vaccine safety and is associated with SEP [37]. Other factors such as occupation are also evident to be associated with the COVID-19 vaccination prevalence [38], which was beyond the scope of our analyses but might have had an influence on our results.

The socioeconomic inequalities in SARS-CoV-2 infection and COVID-19 vaccination are also reflected in the antigen contacts. Our results show a socioeconomic gradient, particularly for the outcome of at least three SARS-CoV-2 antigen contacts. Research suggests that three antigen contacts provide a high protection against severe courses of COVID-19 [39], particularly the combination of SARS-CoV-2 infection and COVID-19 vaccination (“hybrid immunity”) [12]. Against this background, our results indicate that, despite their higher risk of infection during the early stages of the pandemic, socioeconomically disadvantaged groups might still be less protected from severe COVID-19 even after 1 year of the vaccination campaign. Furthermore, the results provide evidence that the socioeconomic gradient regarding the three antigen contacts is attributable less to socially unequal distribution of SARS-CoV-2 infection than to social inequalities in COVID-19 vaccination coverage.

This is the first study from Germany to analyse associations between SEP and SARS-CoV-2 infections, COVID-19 vaccination and antigen contacts after 1 year of the vaccination campaign. One of its major strengths is the seroepidemiological design, which enabled the identification not only of known but also of undetected infections via serological assays. Furthermore, combining the serological data with panel-survey data on socioeconomics enabled differentiated analysis by SEP groups. By assessing the dates of vaccination, we were also able to investigate, the average time to first vaccination by socioeconomic stratification.

Despite these strengths, several limitations should be noted. As our analyses regarding SARS-CoV-2 infections and vaccinations are partly based on self-reports of the participants, recall bias might have occurred, i.e., that the participants did not remember events accurately. This might have led to misreporting of self-reports, either overestimating or underestimating infections or even vaccinations. A further limitation is the fact that the antibody levels may have waned over time and hence contributed to an underestimation of the cumulative infection prevalence, especially among those individuals who had their antigen contacts long before their participation in the study. This concern might also have been relevant regarding vaccinations, where evidence reveals a rapid decline of vaccine effectiveness against SARS-CoV-2 infections but a remaining high protection against severe courses of COVID-19 [40]. Another limitation is that the booster vaccination rates increased strongly during the survey period, which might have limited the interpretation of our results regarding the three antigen contacts. Study participants who participated early in the observation period did not have the opportunity to get a booster vaccination compared to those who participated later, when the booster dose was already available. Considering the longer time to get vaccinated we found for participants with lower education or income, which might also have been the case for the booster vaccination, this might have led to an underestimation of three antigen contacts in these groups. Furthermore, we assume that high-education groups are more likely to participate in surveys than low-education groups, which might introduce self-selection bias and therefore an overrepresentation of higher educated groups in our sample. However, weighting procedures were applied to compensate for systematic non-response and to increase the generalizability of the results for the adult German population in private households [25].

Altogether, our findings suggest socioeconomic inequalities in SARS-CoV-2 infection, COVID-19 vaccination, basic immunization and at least three antigen contacts. These patterns indicate that socioeconomically disadvantaged groups are less protected from severe COVID-19 after almost 2 years of the pandemic and 1 year of the German vaccination campaign, but also that the medium socioeconomic groups still showed potential for prevention. Our results highlight the need for a stronger consideration of socioeconomic determinants in the management of pandemics with infectious diseases to prevent health inequalities. Health policies should consider socioeconomic factors that might influence the risk of infection, barriers to vaccination and vaccine hesitancy. Further research is needed, addressing the reasons for socioeconomic patterning of COVID-19 vaccination in order to design targeted interventions.

## Data Sharing

The data cannot be made publicly available because informed consent from participants did not cover the public deposition of data. However, the data underlying the analysis in this article is archived in the SOEP Research Data Centre (https://www.diw.de/en/diw_01.c.601584.en/data_access.html) in Berlin and can be accessed on site upon reasonable request.
