# Supplementary material for: Socioeconomic Differences in SARS-CoV-2 Infection and Vaccination in Germany: A Seroepidemiological Study After One Year of COVID-19 Vaccination Campaign
Source: Int J Public Health. 2023 Sep 14;68:1606152. doi: 10.3389/ijph.2023.1606152 (PMC10538434; doi:10.3389/ijph.2023.1606152)
Supplement: Supplementary file 1 [file DataSheet1.docx]

SUPPLEMENTARY MATERIAL

**Socioeconomic differences in SARS-CoV-2 infection and vaccination in Germany:** **a seroepidemiological study** **after one year of
COVID-19 vaccination campaign**

**Table supp. 1** Prevalence ratios and confidence intervals of SARS-CoV-2 infection, COVID-19 vaccination, basic immunization and at least three antigen contacts by SEP indicators among adults in Germany, November 2021-March 2022

|  |  | **SARS-CoV-2 infection** | |  | **COVID-19 vaccination^a^** | | |  | **Basic immunization^b^** | | |  | **At least 3**  **antigen contacts** | | |
| --- | --- | --- | --- | --- | --- | --- | --- | --- | --- | --- | --- | --- | --- | --- | --- |
|  |  | PR  (95% CI) | *p* |  | PR  (95% CI) | | *p* |  | PR  (95% CI) | | *p* |  | PR  (95% CI) | | *p* |
| **Education** | |  |  |  |  |  | |  |  |  | |  |  |  | |
|  | Low | 1.35  (1.01,1.82) | 0.045 |  | 0.94  (0.90,0.98) | 0.002 | |  | 0.90  (0.86,0.95) | <0.001 | |  | 0.74  (0.65,0.84) | <0.001 | |
|  | Medium | 1.09  (0.90,1.33) | 0.391 |  | 0.98  (0.96,0.99) | 0.001 | |  | 0.96  (0.94,0.98) | <0.001 | |  | 0.87  (0.81,0.94) | <0.001 | |
|  | High (ref.) | – |  |  | – |  | |  | – |  | |  | – |  | |
| **Income** | |  |  |  |  |  | |  |  |  | |  |  |  | |
|  | Low | 1.09  (0.78,1.52) | 0.604 |  | 0.94  (0.91,0.97) | <0.001 | |  | 0.92  (0.88,0.95) | <0.001 | |  | 0.66  (0.57,0.77) | <0.001 | |
|  | Medium | 1.09  (0.84,1.43) | 0.504 |  | 0.98  (0.97,1.00) | 0.086 | |  | 0.98  (0.96,1.00) | 0.048 | |  | 0.88  (0.81,0.96) | 0.006 | |
|  | High (ref.) | – |  |  | – |  | |  | – |  | |  | – |  | |
| *ref* reference group, *PR* prevalence ratios from Poisson regressions with adjustments for age, sex, migration status, urban–rural residence, federal state, date of participation (and education); ^a^ at least one dose of COVID-19 vaccine; ^b^ due to SARS-CoV-2 infection and/or COVID-19 vaccination | | | | | | | | | | | | | | | |

**Table supp. 2** At least one COVID-19 vaccination by SEP indicators and age group in Germany, November 2021-March 2022

|  |  | | **18-59** | | | | | | **60+** | | | | | |
| --- | --- | --- | --- | --- | --- | --- | --- | --- | --- | --- | --- | --- | --- | --- |
|  |  |  | % (95% CI) | | PR (95% CI) | | *p* | | % (95% CI) | | PR (95% CI) | | *p* | |
| **Education** | | |  | |  | |  | |  | |  | |  | |
| Low | | 86.8 (81.3-90.8) | | 0.92 (0.87-0.97) | | 0.002 | | 95.9 (89.1-98.6) | | 0.98 (0.94-1.02) | | 0.380 | |  |
| Medium | | 91.6 (90.0-93.0) | | 0.97 (0.95-0.99) | | 0.003 | | 97.0 (95.6-97.9) | | 0.99 (0.98-1.01) | | 0.471 | |  |
| High | | 94.5 (92.9-95.8) | | ref. | |  | | 97.3 (95.8-98.2) | | ref. | |  | |  |
| **Income** | | |  | |  | |  | |  | |  | |  | |
|  | Low | | 86.0 (81.8-89.3) | | 0.93 (0.89-0.97) | | <0.001 | | 94.2 (90.6-96.5) | | 0.95 (0.92-0.99) | | 0.005 | |
|  | Medium | | 92.7 (91.1-94.0) | | 0.98 (0.96-1.00) | | 0.058 | | 97.7 (96.6-98.5) | | 0.99 (0.98-1.01) | | 0.267 | |
|  | High | | 96.7 (94.7-98.0) | | ref. | |  | | 98.6 (97.1-99.4) | | ref. | |  | |

*ref* reference group, *%* weighted prevalence, *CI* confidence interval, *PR* prevalence ratios from Poisson regressions with adjustments for age, sex, migration status, urban–rural residence, federal state, date of participation (and education)

**Table supp. 3** Prevalence estimates and prevalence ratios with confidence intervals of at least three antigen contacts by SEP indicators among adults in Germany, November 2021-March 2022

|  |  |  | **At least three contacts  (only vaccine-induced)** | |  | **At least three antigen contacts (hybrid induced through vaccination and infection)** | |
| --- | --- | --- | --- | --- | --- | --- | --- |
|  |  |  | Estimate  (95% CI) | *p* |  | Estimate  (95% CI) | *p* |
| **Education** | | |  |  |  |  |  |
|  | | |  |  |  |  |  |
|  | Prevalence (%)^a^ | |  |  |  |  |  |
|  |  | Low | 22.3  (18.7–26.4) | – |  | 5.4  (3.7–8.0) | – |
|  |  | Medium | 27.5  (25.7–29.4) | – |  | 4.9  (4.2–5.8) | – |
|  |  | High | 30.2  (28.0–32.4) | – |  | 5.1  (4.1–6.3) | – |
|  | Prevalence ratio^b^ | |  |  |  |  |  |
|  |  | Low | 0.68  (0.58–0.79) | <0.001 |  | 1.09  (0.71–1.68) | 0.699 |
|  |  | Medium | 0.85  (0.78–0.92) | <0.001 |  | 0.99  (0.76–1.29) | 0.948 |
|  |  | High (ref.) | – | – |  | – | – |
|  |  |  |  |  |  |  |  |
| **Income** | | |  |  |  |  |  |
|  | | |  |  |  |  |  |
|  | Prevalence (%)^a^ | |  |  |  |  |  |
|  |  | Low | 19.7  (16.7–23.0) | – |  | 4.1  (3.0–5.7) | – |
|  |  | Medium | 27.7  (25.9–29.5) | – |  | 5.4  (4.6–6.3) | – |
|  |  | High | 34.6  (31.5–37.9) | – |  | 4.3  (3.3–5.8) | – |
|  | Prevalence ratio^b^ | |  |  |  |  |  |
|  |  | Low | 0.59  (0.50–0.70) | <0.001 |  | 0.87  (0.56–1.35) | 0.535 |
|  |  | Medium | 0.82  (0.75–0.91) | <0.001 |  | 1.14  (0.81–1.59) | 0.461 |
|  |  | High (ref.) | – |  |  | – | – |
|  |  |  |  |  |  |  |  |

^a^ weighted prevalence; ^b^ *ref* reference group, adjusted for age, sex, migration status, urban–rural residence, federal state, date of participation (and education); CI confidence interval
